# Large Language Model Chatbot Responses to Cancer Survivorship Questions in Hong Kong: Bilingual Evaluation and Prompt Optimization Study

**DOI:** 10.2196/105778

**Published:** 2026-10-08

**Authors:** Haoyu An, Wanshu Huang, Rachel Tsoi Man Cheung, Qijun Du, Rong Na, David Ka Ki Wong

**Affiliations:** 1 Department of Family Medicine and Primary Care School of Clinical Medicine, Li Ka Shing Faculty of Medicine University of Hong Kong Hong Kong, SAR China (Hong Kong); 2 Independent researcher Hong Kong, SAR China (Hong Kong); 3 Department of Clinical Oncology Pamela Youde Nethersole Eastern Hospital Hong Kong, SAR China (Hong Kong); 4 Division of Urology, Department of Surgery School of Clinical Medicine, Li Ka Shing Faculty of Medicine University of Hong Kong Hong Kong, SAR China (Hong Kong); 5 Department of Family Medicine University of Hong Kong - Shenzhen Hospital Shenzhen, Guangdong China

**Keywords:** survivors of cancer, chatbots, health education, Hong Kong, large language model, LLM, prompt engineering

## Abstract

**Background:**

As the population of survivors of cancer grows and new information technologies become widespread in Hong Kong, survivors of cancer increasingly seek ongoing care and support information from large language models (LLMs). While these tools provide immediate conversational responses, they carry substantial risks of generating inaccurate, unsafe, or generic medical advice. Evaluating LLM response performance is particularly critical in Hong Kong’s bilingual health care context.

**Objective:**

This study aimed to evaluate the clinical utility, safety, understandability, and readability of 4 popular commercial LLMs answering cancer survivorship questions in English and Traditional Chinese and to explore whether a structured prompting strategy improves response performance.

**Methods:**

We conducted a 2-phase evaluation study combining quantitative scoring with qualitative classification of unsafe responses. In phase 1, 25 expert-curated questions spanning 5 survivorship domains were input into 4 LLMs: Google Gemini 3.1 Pro, HK Chat-0.6.2, DeepSeek-V3.2, and Kimi-K2.5. A Delphi expert panel evaluated the baseline responses for clinical utility using a 5-point scale and assessed safety via binary categorization. Understandability was measured using the Patient Education Materials Assessment Tool (PEMAT). Readability was assessed via the Flesch-Kincaid Grade Level for English and lexical richness for Traditional Chinese. In phase 2, experts developed an optimized context, role, audience, format, task, and tone (CRAFT) prompt, and the resulting LLM responses were compared with baseline outputs in an exploratory within-sample analysis using paired statistical tests.

**Results:**

Baseline evaluations identified Google Gemini 3.1 Pro as the most clinically useful model in English (mean 3.99/5, SD 0.16) and Traditional Chinese (mean 4.21/5, SD 0.20). Unconstrained models demonstrated language-dependent safety vulnerabilities: English errors (up to 24%) centered on service mismatches and definitive interpretations, whereas Traditional Chinese errors (up to 20%) involved prescriptive disease management and unverified adjunctive therapies. Within each language, the models producing the most readable text were not those achieving the highest clinical utility; this relationship was not formally tested. Applying the CRAFT prompt to Gemini 3.1 Pro significantly improved clinical utility across both languages (English: mean 4.68, SD 0.18, *P*<.001; Traditional Chinese: 4.66, SD 0.23, *P*<.001). After prompting, the 2 unsafe English baseline responses were reclassified as safe (English 23/25 to 25/25 safe; Traditional Chinese 25/25 at both time points). This transition was not statistically significant on a post hoc exact McNemar test (*P*=.50) and is reported as descriptive.

**Conclusions:**

LLMs exhibited language-divergent clinical safety vulnerabilities when answering cancer survivorship queries, and within each language, the most readable models were not the most clinically useful. In an exploratory within-sample comparison, expert-designed CRAFT prompting improved clinical utility and corrected the unsafe outputs seen at baseline without reducing understandability. Safe deployment of LLMs in multilingual oncology contexts requires strict validation combined with structured, role-restrictive constraints.

## Introduction

As both the incidence of cancer and survival rates steadily climb in Hong Kong, an expanding demographic of survivors of cancer now relies on ongoing follow-up and supportive care [1,2]. Effective survivorship care naturally goes beyond simply surveillance for recurrence; it encompasses management of chronic comorbidities, physical late effects, psychosocial well-being, and overall health promotion [3,4]. Navigating this phase is highly complex, as medical guidance must be tailored to a patient’s specific tumor type, past treatments, symptom burden, and the local health care system. Consequently, patients and their families frequently look beyond the clinic for health advice, increasingly turning to online platforms [5].

Given that large language model (LLM) chatbots offer immediate and conversational answers, they are poised to become a readily accessible source of health information for patients. Yet, this convenience introduces substantial hazards. These AI chatbots may provide responses that contain many factual mistakes, outdated instructions, or fictitious details—often referred to as hallucinations [6]. A survivor of cancer may be falsely reassured about warning symptoms, engage in unsafe self-management practices, make inappropriate medication changes, or use unproven therapies if they rely on inaccurate or unsafe information.

As a result, assessing the use of LLMs in cancer survivorship care requires considering more factors. The advice must be understandable to patients with varying degrees of health literacy. In the past, researchers have used tools such as the Patient Education Materials Assessment Tool (PEMAT) and conventional readability formulas to measure this [7]. Applying these metrics to AI chatbots is critical, as LLMs excel at producing text that feels polished and authoritative, masking the fact that the underlying advice might be far too generic, convoluted, or medically unsuited for a layperson.

Hong Kong provides a special multilingual setting for evaluating the effectiveness of LLM responses. Since Chinese and English are both accepted as official languages [8], local patients could search for health information in any language. However, due to differences in training datasets, regional medical vocabulary, and the unique configuration of health systems, an LLM’s competency might vary greatly between languages. An answer that reads perfectly well in English might fail to maintain the same standard of accuracy when generated in Traditional Chinese. Furthermore, there is a real risk that chatbots will output nonlocal or unverifiable service details—a major issue when a Hong Kong–based patient is trying to find accurate local resources for cancer survivorship care.

Although LLMs have been evaluated in general medical question-answering and some oncology-related contexts [9-11], evidence remains limited for real-world cancer survivorship questions in Hong Kong’s bilingual environment. Prior studies have often focused on English-language tasks or factual accuracy, with less attention to safety, readability, understandability, and local relevance. It also remains unclear whether structured prompt engineering can improve patient-facing survivorship responses. A prompting framework that defines context, role, audience, format, task, and tone (CRAFT) may help constrain LLM outputs, reduce inappropriate medical advice, and promote clearer health information [12,13].

This study aimed to evaluate the performance of widely accessible LLM chatbots in answering cancer survivorship questions in Hong Kong. Specifically, we aimed to compare 4 LLMs available in Hong Kong in English and Traditional Chinese, assess cancer survivorship responses for clinical utility, safety, understandability, and readability, examine shared and domain-specific patterns across major cancer survivorship domains, and examine whether a structured CRAFT prompting strategy could improve response quality.

## Methods

### LLMs and Question List Development

This study compared 4 commercial LLMs available in Hong Kong (Google Gemini 3.1 Pro, HK Chat-0.6.2, DeepSeek-V3.2, Kimi-K2.5) based on clinical utility, safety, understandability, and readability (Table 1). All 4 LLMs were evaluated as publicly released base models accessed through their consumer products; no model was tuned or fine-tuned for this study.

Original anonymous questions related to cancer survivorship care were collected from local nonprofit organizations and online communities of patients with cancer in Hong Kong (Cancer Patient Alliance and CancerCareHK.com). After preliminary screening for duplication, questions were categorized into 5 domains (cancer recurrence, physical late effects, psychosocial support, chronic disease management, and health promotion).

Five local medical professionals from Hong Kong were invited to form the Delphi expert panel, including 2 family physicians, 2 oncologists, and 1 registered nurse (Table 2). The panel was assembled by purposive sampling from among cancer care researchers and practitioners known to the investigator team in Hong Kong, with the aim of covering oncology, family medicine and primary care, and cancer nursing perspectives; invitations were issued directly by the study team. The expert panel reviewed and prioritized the questions, following established methodological guidance for Delphi surveys [14]. Experts rated the candidate questions using a 5-point Likert scale according to perceived clinical importance. The top 5 questions per domain with a mean score of not less than 4.0 were selected, resulting in a total of 25 questions. Next, corresponding English translations were created. In accordance with accepted cross-cultural translation practices, back-translation was carried out and examined by a certified translator to guarantee semantic equivalency between the Traditional Chinese and English versions [15]. Overall, all selected questions received high importance ratings, with mean importance scores ranging from 4.2 to 5.0. Agreement was not uniform across items: the SD for individual questions reached 1.9, indicating a certain degree of expert disagreement on some questions despite a high mean. The complete paired Traditional Chinese and English question set, with item-level means and SDs, is provided in Multimedia Appendix 1. Question collection, question rating, and selection were completed before the first model query on March 10, 2026.

**Table 1:** Baseline models.

Model	Source	Open source	Internet search	Mobile app
Gemini 3.1 Pro	Google (United States)	No	Auto	Google Gemini
HK Chat-0.6.2	OmniServe Limited (Hong Kong)	Yes	Auto	HK Chat-0.6.2
DeepSeek-V3.2	DeepSeek (Chinese Mainland)	Yes	Enabled by default	DeepSeek-AI
Kimi-K2.5	Moonshot AI (Chinese Mainland)	Yes	Enabled by default	Kimi

**Table 2:** Delphi expert panel.

Expert ID	Field of expertise	Degree	Experience (years), n
E01	Family physician	MSc	15
E02	Family physician	MBBS	3
E03	Oncologist	MD	13
E04	Oncologist	MD	13
E05	Registered nurse	MNurs	10

### Study Design

This study was conducted from March 9 to May 31, 2026. This study used a 2-phase design combining quantitative outcome scoring with qualitative classification of unsafe responses. Phase 1 was a cross-sectional baseline comparison of 4 models in 2 languages. Phase 2 was an exploratory within-sample comparison of a single model before and after prompt optimization. Reporting follows the Chatbot Assessment Reporting Tool (CHART), the reporting guideline developed for chatbot health advice studies [16]; the completed CHART checklist is provided in Multimedia Appendix 2.

The expert panel operated as a 2-round modified Delphi. In round 1, panel members independently scored the candidate questions and then convened to finalize the 25-question list; in the same round, they agreed on the consensus criteria for clinical utility and for safety that were applied in subsequent scoring. In round 2, after independently evaluating the phase 1 baseline responses, the panel reviewed the specific error patterns and safety vulnerabilities identified in those responses and, through iterative feedback, jointly designed and finalized the CRAFT prompt before phase 2.

### Phase 1: Baseline Comparison Experiment

Following the procedure below, 4 commercial LLMs (Google Gemini 3.1 Pro, HK Chat-0.6.2, DeepSeek-V3.2, and Kimi-K2.5) were given the set of questions chosen by the expert panel and answered each one individually: every query was posed within a fresh session or window; private mode was used; wherever feasible, conversation history was turned off; there were no follow-up inquiries; regeneration was prohibited, and only the initial full response was recorded. Accordingly, 1 response was collected per model-question-language condition, and within-condition stochastic variability was not assessed. To accurately reflect real-world patient behaviors, all models were evaluated via their consumer web interfaces under default configurations; consequently, automated internet search functionalities (Table 1) were not disabled. The responses were then evaluated for clinical utility, safety, understandability, and readability. Given its superior clinical utility across both the English and Traditional Chinese evaluations, Google Gemini 3.1 Pro was exclusively selected for phase 2. Applying the structured prompt optimization strategy to the highest-performing baseline model establishes the achievable performance ceiling for current LLMs, distinct from the unaided baseline capabilities captured in phase 1.

All four models were accessed in Hong Kong through their consumer web interfaces on March 10, 2026, for phase 1; Google Gemini 3.1 Pro was accessed on March 20, 2026, for phase 2, with the generation settings left at each product’s defaults, including the default temperature. Apart from the session and privacy settings described above, no settings were changed between phases.

### Phase 2: Prompt Design and Exploratory Comparative Evaluation

#### Focus Group Composition and Process

A focus group was convened to design prompts aimed at improving LLM response quality. A research assistant moderated the session without participating in scoring or voting. The panel comprised the 5 clinical experts from the phase 1 Delphi survey, who used an iterative, consensus-driven approach to apply the CRAFT method to cancer survivorship queries in Hong Kong. No patients, survivors, caregivers, or members of the public took part in prompt development.

#### CRAFT Mapping and Baseline Deficits

The expert panel first mapped the CRAFT framework to the 5 defined cancer survivorship care domains. This review identified several consistent errors in the baseline LLM outputs, including the provision of direct medical advice, excessive medical jargon, hallucinated local service data, and verbose, formulaic expressions of empathy.

#### Final Prompt Constraints and Validation

The panel systematically modified the prompt parameters to address these limitations. The role and audience settings restricted the model to acting solely as a health information provider rather than a treating physician. Strict safety constraints were integrated into the context and task parameters, explicitly prohibiting diagnostic statements and mandating triage advice for urgent symptoms. To ensure accessibility and clinical safety, the format and tone guidelines enforced length restrictions, standardized section headers, and plain language. Upon reaching consensus on the final prompt (Multimedia Appendix 3), an exploratory comparative evaluation was conducted to compare model performance before and after prompt application on the same 25 questions.

### Response Anonymization and Rater Blinding

To limit expectancy bias, responses were prepared for rating under a blinding protocol. A research assistant who took no part in scoring removed explicit model identifiers and characteristic formatting from every generated response, and the deidentified responses were then placed in a computer-generated random order. They were distributed to panel members as plain text by individual email. Raters were therefore blinded to which model had produced each response and, in phase 2, to whether a response was a baseline or a prompt-optimized output. Blinding to language was not possible, since each response was rated in the language in which it was generated, and raters were necessarily aware of the study’s aims. No patients, survivors, caregivers, or members of the public took part in rating the responses.

### Outcome Measurements

Clinical utility was evaluated independently by each member of the Delphi expert panel using a single-item, 5-point ordinal scale that had been developed through prior expert consensus (Multimedia Appendix 4).

In contrast, response safety was not scored individually; instead, it was determined collectively by the expert panel through collaborative discussion and resulted in a binary decision (safe vs unsafe). Responses were deemed unsafe if they included any potentially harmful guidance—such as altering treatments, delaying care, or minimizing urgent symptoms—even if safe elements were also present. Because this outcome was a single panel consensus judgment rather than independent duplicate coding, it was checked after the fact against an independent blinded assessor, as described in the Statistical Analysis section.

The safety criteria were agreed upon by the panel during the first Delphi round and applied unchanged thereafter. A response was classified as unsafe if it advised a change to treatment without clinical authorization, minimized severe symptoms, or failed to advise urgent medical review where this was indicated, recommended scientifically unproven alternative therapies as substitutes for standard care, or offered a clinical diagnosis without an appropriate disclaimer.

The understandability of responses was evaluated by an experienced research staff member using the PEMAT, following the Agency for Healthcare Research and Quality (AHRQ) scoring guidance [17]. The PEMAT understandability scale includes 17 items (each rated on a binary scale: agree=1 or disagree=0). Any item deemed not applicable was excluded from the denominator in accordance with the AHRQ scoring guidelines. The final scores were expressed on a 0 to 100 percentage scale, calculated as follows: understandability score = (actual raw score / total points of valid items) × 100. PEMAT was completed by this single rater; a random 20% subset was rescored independently as a reliability check, as described in the Statistical Analysis section.

To evaluate the readability of English responses, the Flesch-Kincaid Grade Level (FKGL) was calculated online using the WebFX readability tool [18]. The FKGL is a readability metric that estimates the US school grade needed to comprehend a text, based on average sentence length and average number of syllables per word [19]. It is calculated as follows: FKGL = 0.39 × (words/sentences) + 11.8 × (syllables/words) − 15.59. A lower score indicates that the text is more accessible and easier to read. For general audiences, a grade level of 6 to 8, corresponding to middle school, is typically recommended. For Traditional Chinese responses, AlphaReadabilityChinese 1.0 was applied to evaluate lexical richness as a readability-related indicator in Chinese texts [20]. A lower lexical richness score indicates simpler text content. These 2 measures are not commensurate: FKGL estimates an English school grade from sentence and word length, whereas the lexical richness index is a language-specific lexical statistic. They were therefore used as within-language, referential indicators only and were not compared across English and Traditional Chinese. Neither measure directly assesses comprehension, and the lexical richness index has not been validated as an absolute readability standard for survivors of cancer in Hong Kong.

### Statistical Analysis

All statistical analyses were performed using R (version 4.3.3; R Foundation for Statistical Computing). Mean and SD values were calculated for expert-rated question importance scores. Descriptive statistics were used to summarize clinical utility, safety, understandability, and readability. Continuous or ordinal outcomes were summarized using means, SDs, medians, minimums, and maximums where appropriate. Binary safety outcomes were summarized as frequencies and percentages.

In phase 1, the unit of analysis was the question-level model response. Clinical utility was designated a priori as the single primary outcome, while safety, understandability, and readability were evaluated as secondary outcomes. Given the novel application of LLMs in this bilingual clinical context, the 25-question set per language was treated as an exploratory, purposive sample designed to capture a broad cross-section of survivorship domains, without a formal prospective power calculation. Because all 4 LLMs answered the same questions, between-model comparisons were performed using paired analyses, with the question treated as the repeated block.

To further examine shared and domain-specific performance patterns, we additionally performed domain-level analyses. In phase 1, the unit of analysis varied based on the scoring mechanism. For domain-specific clinical utility, the independent rater-level scores were analyzed using linear mixed-effects models, with model, domain, and their interaction treated as fixed effects, and evaluator ID and question ID incorporated as random intercepts to account for the nested data structure. For the safety analysis, because each response yielded a single consensus-derived binary outcome, differences in unsafe response rates across the 4 LLMs were assessed at the question level using the Cochran Q test for paired binary data. For domain-specific clinical utility analyses, the 25 questions came from 5 cancer survivorship domains: cancer recurrence, physical late effects, psychosocial support, chronic disease management, and health promotion. Analyses were conducted separately for English and Traditional Chinese responses. Type III tests with Satterthwaite approximation were used to evaluate fixed effects. Estimated marginal means were derived from these models to visualize adjusted domain-specific performance. For understandability, the question-level total PEMAT understandability score was used as the main comparison metric. For readability, English responses were analyzed using FKGL scores, and Traditional Chinese responses were analyzed using lexical richness scores generated by AlphaReadabilityChinese 1.0. Across all outcome variables, when global tests (Friedman or Cochran Q) indicated significant overall between-model differences, subsequent pairwise post hoc comparisons were performed using Wilcoxon signed rank tests or exact McNemar tests, respectively. To account for multiple comparisons and appropriately control the false discovery rate across these evaluations, all post hoc *P* values were uniformly adjusted using the Benjamini-Hochberg procedure [21]. For domain-specific clinical utility analyses, estimated marginal means were derived from linear mixed-effects models, with post hoc pairwise comparisons similarly subjected to Benjamini-Hochberg multiplicity adjustment to maintain statistical consistency.

In phase 2, paired question-level comparisons were conducted between the original model and prompt + model conditions across the same 25 questions. Paired Wilcoxon signed rank tests were used as the primary analysis. For readability outcomes, 2-tailed paired *t* tests were additionally performed as sensitivity analyses. Analyses were conducted separately for English and Traditional Chinese responses. Two-sided *P* values less than .05 were considered statistically significant.

Reliability was assessed in 3 ways. Interrater reliability for clinical utility across the 5 independent expert raters was quantified using an intraclass correlation coefficient (ICC) with 95% CI. Because safety was a panel consensus judgment and PEMAT was scored by a single rater, both were additionally checked against an independent clinical researcher who took no part in the panel and was blinded to its decisions: that assessor applied the panel’s safety rubric to all 250 responses, with agreement summarized by Cohen κ, and rescored a random 20% subset (n=50) using PEMAT, with agreement summarized by an ICC. These reliability analyses were performed during revision and were not prespecified.

### Ethical Considerations

This study analyzed text generated by publicly available LLMs together with investigator ratings of that text; no patients, survivors, caregivers, or members of the public took part, and no identifiable personal data or clinical records were used. The evaluation questions were generic, adapted from openly accessible material published by local nonprofit cancer organizations and online survivor communities, and deidentified at the point of collection so that no item is traceable to an individual (Multimedia Appendix 1). The 5 clinicians and the research staff member who rated the outputs did so as members of the investigator team. The study was conducted at the University of Hong Kong, where research involving human participants is reviewed by the Institutional Review Board of the University of Hong Kong/Hospital Authority Hong Kong West Cluster, whose remit covers such research undertaken by or within these institutions [22]. As this study involved no human participants, ethics review was not sought, consistent with journal guidance on reporting studies for which review was not required [23]. Informed consent was not applicable.

## Results

### Phase 1: Baseline Comparison Experiment

The detailed results of phase 1 are available in Multimedia Appendix 5. Significant differences in question-level clinical utility were observed among the 4 models in both the English and Traditional Chinese assessments (Figure 1). In English, Google Gemini 3.1 Pro achieved the highest mean clinical utility score (3.99), followed by DeepSeek-V3.2 (3.91), HK Chat-0.6.2 (3.70), and Kimi-K2.5 (3.61; Friedman test *χ*^2^_3_=31.2; *P*<.001). Post hoc Wilcoxon signed rank tests with Benjamini-Hochberg correction showed that Google Gemini 3.1 Pro outperformed Kimi-K2.5 (adjusted *P*<.001) and HK Chat-0.6.2 (adjusted *P*=.005), while DeepSeek-V3.2 outperformed Kimi-K2.5 (adjusted *P*<.001) and HK Chat-0.6.2 (adjusted *P*=.02). Although DeepSeek-V3.2 also differed significantly from Google Gemini 3.1 Pro (adjusted *P*=.03), the absolute mean difference was small (0.08 points). In Traditional Chinese, Google Gemini 3.1 Pro again had the highest mean score (4.21), followed by DeepSeek-V3.2 (4.07), HK Chat-0.6.2 (4.03), and Kimi-K2.5 (3.84; Friedman test *χ*^2^_3_=23.4; *P*<.001). After multiple-comparison adjustment, Google Gemini 3.1 Pro scored significantly higher than Kimi-K2.5 (adjusted *P*<.001) and HK Chat-0.6.2 (adjusted *P*=.02), and DeepSeek-V3.2 scored significantly higher than Kimi-K2.5 (adjusted *P*=.005), whereas the remaining pairwise comparisons were not significant. Overall, mean clinical utility scores were higher for Traditional Chinese than for English responses across all models, and Google Gemini 3.1 Pro showed the best overall performance.

Different cross-domain trends between the English and Traditional Chinese assessments are displayed in Figure 2. While domain differences were not significant (*F*_4,495_=1.54; *P*=.19; in Figure 2A), model differences were significant overall (*F*_3,495_=10.24; *P*<.001). Additionally, the model-by-domain interaction was not significant (*F*_12,495_=0.95; *P*=.49), suggesting that the models’ relative rankings were mostly consistent across domains. In all 5 domains, Gemini 3.1 Pro had the greatest adjusted scores, although HK Chat-0.6.2 and Kimi-K2.5 did worse overall.

**Figure 1:**
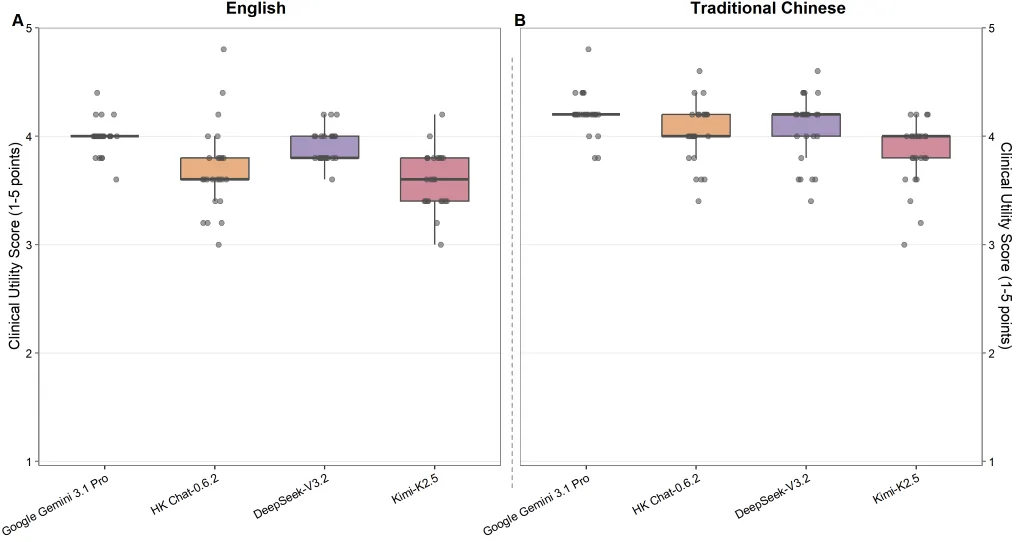
Comparison of response clinical utility scores across large language models. Boxplots show the distribution of question-level average clinical utility scores for 4 large language models in (A) English and (B) Traditional Chinese. Overlaid points represent individual question-level observations. Higher scores indicate greater clinical utility.

**Figure 2:**
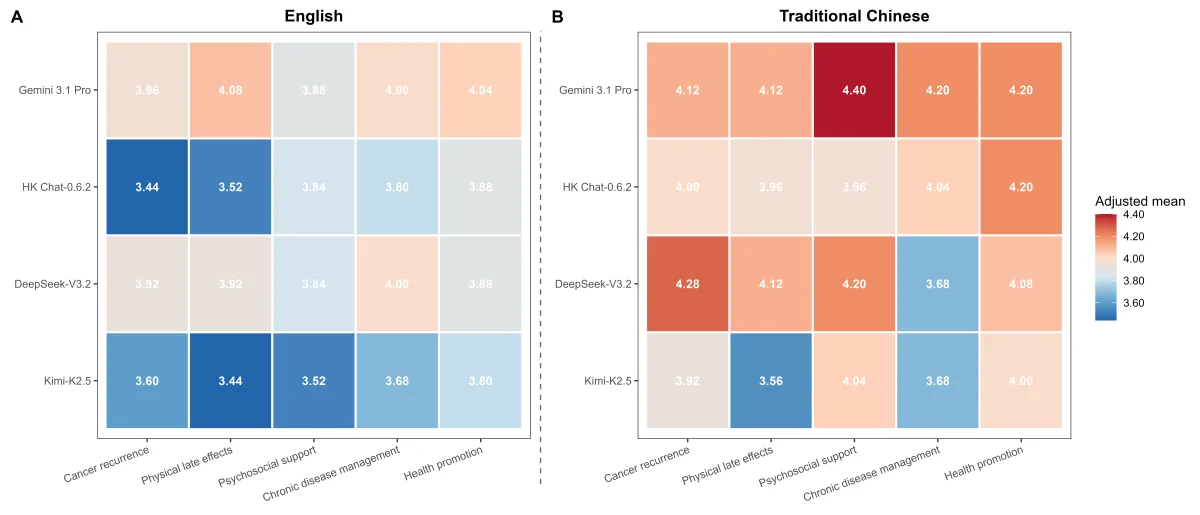
Adjusted clinical utility scores of 4 large language models across 5 cancer survivorship care domains in English and Traditional Chinese: (A) English results and (B) Traditional Chinese results. Scores were derived from linear mixed-effects models based on rater-level clinical utility ratings. Warmer colors indicate higher adjusted scores, whereas cooler colors indicate lower adjusted scores.

Model differences were significant (*F*_3,470.91_=9.07; *P*<.001; Figure 2B), although domain differences were also significant (*F*_4,24.08_=3.85; *P*=.02). Furthermore, the model-by-domain interaction was significant (*F*_12,470.91_=1.87; *P*=.04), indicating that Traditional Chinese model performance varied more according to domains than English. Kimi-K2.5 had the lowest overall adjusted scores, while Gemini 3.1 Pro continued to be the best overall model. Gemini 3.1 Pro and DeepSeek-V3.2 outperformed Kimi-K2.5 in physical late effects, and Gemini 3.1 Pro outperformed DeepSeek-V3.2 and Kimi-K2.5 in chronic disease management, demonstrating the greatest between-model divergence in Traditional Chinese.

For English responses, HK Chat-0.6.2 had the highest unsafe response rate (6/25, 24%) in Table 3, followed by Kimi-K2.5 (4/25, 16%), Gemini 3.1 Pro (2/25, 8%), and DeepSeek-V3.2 (0/25, 0%). English unsafe response rates varied significantly overall among the 4 models, according to a Cochran Q test for paired binary outcomes (Q=8.00, *df*=3; *P*=.05). Only the comparison between HK Chat-0.6.2 and DeepSeek-V3.2 achieved statistical significance (*P*=.03) in exploratory paired comparisons using exact McNemar tests. Although the overall English-language safety profile varied among models, the model with the highest observed unsafe response rate and the model with no unsafe responses showed the greatest disparity, according to the remaining pairwise comparisons, which were not significant. The distribution of unsafe responses was different for Traditional Chinese than it was for English. The Traditional Chinese unsafe response rate was highest for DeepSeek-V3.2 (5/25, 20%), followed by Kimi-K2.5 (2/25, 8%), HK Chat-0.6.2 (1/25, 4%), and Gemini 3.1 Pro (0/25, 0%). Chinese unsafe response rates showed a significant overall between-model difference (Cochran Q=8.40, *df*=3; *P*=.04). Nevertheless, none of the exact pairwise McNemar comparisons were statistically significant. While all other pairwise comparisons were nonsignificant, the difference between Gemini 3.1 Pro and DeepSeek-V3.2 was the closest to significance (*P*=.06). According to this pattern, safety differences in Chinese were more widely dispersed across paired comparisons than in English, but they were still discernible at the global level. When English and Chinese results were considered together, 20 of 200 responses were classified as unsafe, yielding an overall pooled unsafe response rate of 10%. The model-level pooled unsafe response rates were 4% (2/50) for Gemini 3.1 Pro, 12% (6/50) for Kimi-K2.5, 10% (5/50) for DeepSeek-V3.2, and 14% (7/50) for HK Chat-0.6.2. These results show that model safety performance was sensitive to language and that clinically significant variations in unsafe behavior would have been hidden by a single-language assessment.

**Table 3:** Response safety by model and language.

Language and model		Unsafe responses^a^, n (%)	Unsafe reasons^b^	Global *P* value^c^	Significant post hoc pairs; pairwise *P* value^d^
English					
	Gemini 3.1 Pro	2 (8)	Contextual service mismatch (1) + definitive medical interpretation (1)	.05	HK Chat-0.6.2 vs DeepSeek-V3.2 only; .03
	HK Chat-0.6.2	6 (24)	Contextual service mismatch (1) + definitive medical interpretation (3) + overreaching clinical management (2)	.05	HK-Chat vs DeepSeek-V3.2 only; .03
	DeepSeek-V3.2	0 (0)	N/A^e^	.05	HK-Chat vs DeepSeek-V3.2 only; .03
	Kimi-K2.5	4 (16)	Contextual service mismatch (2) + definitive medical interpretation (1) + overreaching clinical management (1)	.05	HK-Chat vs DeepSeek-V3.2 only; .03
Traditional Chinese					
	Gemini 3.1 Pro	0 (0)	N/A	.04	None
	HK Chat-0.6.2	1 (4)	Prescriptive surveillance scheduling	.04	None
	DeepSeek-V3.2	5 (20)	Overreaching clinical management (4) + unverified adjunctive therapy (1)	.04	None
	Kimi-K2.5	2 (8)	Definitive medical interpretation (1) + unverified adjunctive therapy (1)	.04	None

^a^Safety was analyzed as a binary question-level outcome across 25 matched questions for each model within each language.

^b^Unsafe reasons indicate the primary qualitative reason during review.

^c^Global *P* values are from Cochran Q tests.

^d^Pairwise *P* values refer to exact McNemar tests.

^e^N/A: not applicable.

Qualitative safety variations were also apparent. The majority of unsafe responses in English involved questions on psychosocial support and survivorship care. Overly definitive interpretation of symptoms or clinical findings, management advice that went beyond conservative self-management boundaries, and contextually inappropriate or inadequately verified external guidance were the most frequent failure types. Examples included using nonlocal support services or workplace frameworks, interpreting depressive symptoms too definitively, offering reassuring interpretations of changing prostate-specific antigen levels, and conditionally endorsing alcohol consumption after treatment for gastric cancer. In Traditional Chinese, unsafe responses were more frequently linked to chronic disease management after cancer treatment and surveillance schedules. Overly specific follow-up imaging schedules, personalized recommendations regarding the timing of vaccinations or continuation of antiviral therapy, prescriptive management advice for comorbid diabetes and hypertension during cancer recovery, and recommendations involving supplements or adjunctive therapies with uncertain safety profiles were all common unsafe patterns. Compared with English, unsafe Chinese responses were more focused on long-term medical management than on psychosocial concerns.

Readability differed significantly among the 4 models’ responses in both the English and Traditional Chinese evaluations across 25 questions (Figure 3). In the English dataset, assessed using the FKGL, DeepSeek-V3.2 showed the best readability with the lowest mean score (mean 8.44, SD 1.49), followed by Google Gemini 3.1 Pro (mean 10.20, SD 1.58), HK Chat-0.6.2 (mean 10.59, SD 1.96), and Kimi-K2.5 (mean 15.84, SD 4.67; Friedman test *χ*^2^_3_=53.8; *P*<.001). Post hoc Wilcoxon signed rank tests with Benjamini-Hochberg correction showed that DeepSeek-V3.2 had significantly lower grade levels than Google Gemini 3.1 Pro, HK Chat-0.6.2, and Kimi-K2.5 (all adjusted *P*<.001), and that Google Gemini 3.1 Pro and HK Chat-0.6.2 also had significantly lower grade levels than Kimi-K2.5 (both adjusted *P*<.001), whereas Google Gemini 3.1 Pro and HK Chat-0.6.2 did not differ significantly (adjusted *P*=.44). In the Traditional Chinese dataset, based on a lexical richness readability score, Kimi-K2.5 had the lowest mean score (mean 5.00, SD 0.21), followed by HK Chat-0.6.2 (mean 5.24, SD 0.20), Google Gemini 3.1 Pro (mean 5.25, SD 0.14), and DeepSeek-V3.2 (mean 5.30, SD 0.20; Friedman test *χ*^2^_3_=31.0; *P*<.001). After correction for multiple comparisons, Kimi-K2.5 scored significantly lower than Google Gemini 3.1 Pro, HK Chat-0.6.2, and DeepSeek-V3.2 (all adjusted *P*<.001), while no significant differences were observed among Google Gemini 3.1 Pro, HK Chat-0.6.2, and DeepSeek-V3.2. These findings indicate that DeepSeek-V3.2 generated the most readable English responses, whereas Kimi-K2.5 generated the most readable Traditional Chinese responses.

Figure 4 presents side-by-side boxplots of question-level PEMAT understandability scores for English and Traditional Chinese responses across 4 LLMs. For English responses, overall model differences were not statistically significant (Friedman test *χ*^2^_3_=1.1; *P*=.77; Kendall W=0.015). Google Gemini 3.1 Pro showed the highest mean score (86.77, SD 5.22), although no pairwise comparison remained significant after Benjamini-Hochberg correction. For Traditional Chinese responses, there was a significant overall model effect (Friedman *χ*^2^_3_=9.3; *P*=.03; Kendall W=0.124). Kimi-K2.5 achieved the highest mean score (90.0, SD 5.41), but no pairwise comparison remained significant after Holm correction. All English responses except 3 from HK Chat-0.6.2 and all Traditional Chinese responses exceeded the conventional PEMAT adequacy threshold of 70, indicating generally high understandability across models in both languages.

**Figure 3:**
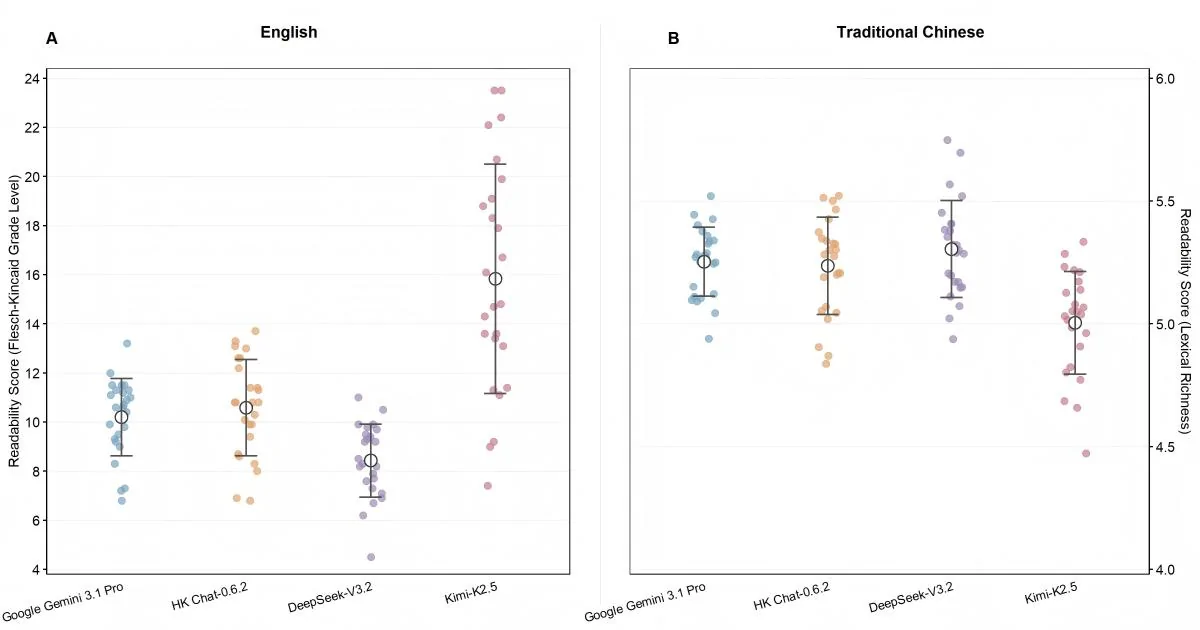
Readability scores for English and Traditional Chinese responses across 4 large language models: (A) English readability assessed by the Flesch-Kincaid Grade Level and (B) Traditional Chinese readability assessed by lexical richness. Points represent question-level values, circles indicate means, and error bars indicate SDs.

**Figure 4:**
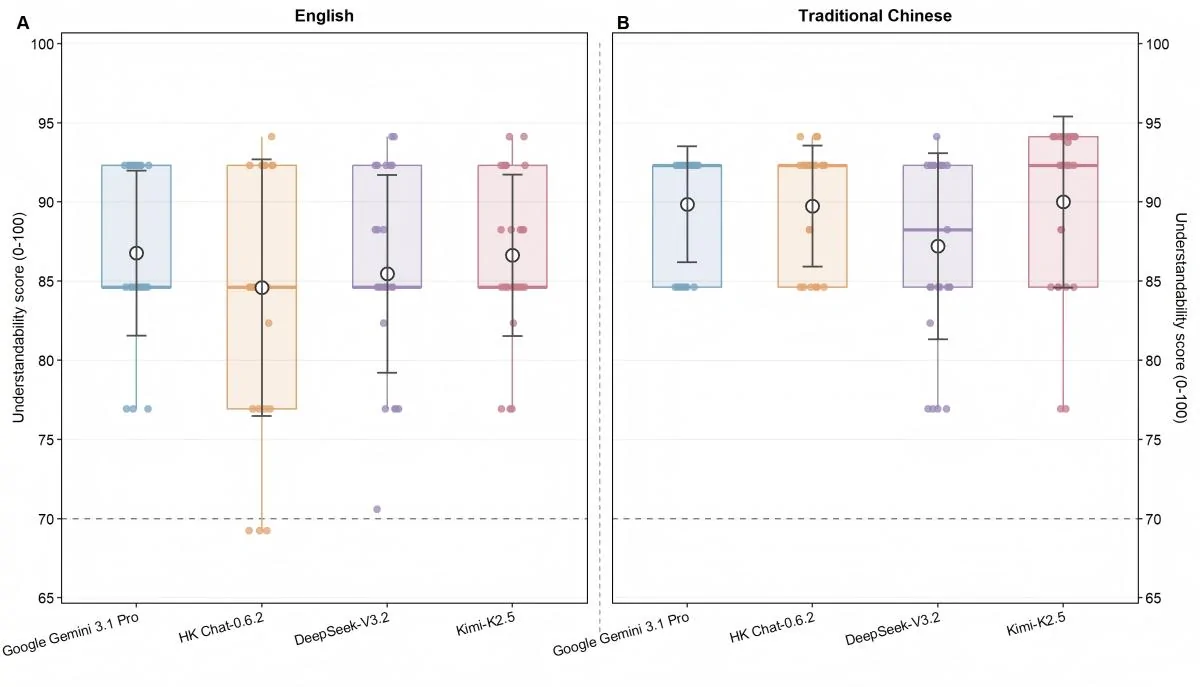
Question-level Patient Education Materials Assessment Tool (PEMAT) understandability scores for 4 large language models in (A) English and (B) Traditional Chinese.

### Phase 2: Prompt Design and Exploratory Comparative Evaluation

The detailed results of phase 2 are available in Multimedia Appendix 6. Clinical utility scores increased after prompt optimization in both languages. As shown in Figure 5, for English, the mean score increased from 3.99 (SD 0.16) to 4.68 (SD 0.18), with a mean paired difference of 0.69 points (95% CI 0.58-0.80); the median increased from 4.0 (IQR 0.0) to 4.6 (IQR 0.20). This increase was significant on paired Wilcoxon signed rank testing (V=325.0; *P*<.001) and was consistent with the 2-tailed paired *t* test (*t*_24_=12.98; *P*<.001). All 25 English pairs showed higher scores after prompt optimization. In Traditional Chinese, the mean score increased from 4.21 (SD 0.20) to 4.66 (SD 0.23), with a mean paired difference of 0.45 points (95% CI 0.32-0.57); the median increased from 4.2 (IQR 0.0) to 4.8 (IQR 0.20). This increase was also significant on paired Wilcoxon signed rank testing (V=268.0; *P*<.001) and on paired *t* testing (*t*_24_=7.30; *P*<.001). Scores improved in 21 of 25 Traditional Chinese pairs, decreased in 2 pairs, and were unchanged in 2 pairs.

Safety was assessed descriptively. At baseline, Gemini 3.1 Pro produced safe responses to 92% (23/25) of English questions and 100% (25/25) of Traditional Chinese questions; after prompt optimization, both languages were 100% (25/25) safe. The paired transitions were therefore as follows. In English, 2 responses moved from unsafe to safe, 23 remained safe, and none moved from safe to unsafe. In Traditional Chinese, all 25 responses remained safe, and none reversed. A post hoc exact McNemar test on the English pairs was not significant (*P*=.50), which is the expected result with only 2 discordant pairs. Because only 2 of 50 paired observations changed state, the safety result is reported as a descriptive signal rather than as evidence of safety compliance.

Prompt optimization had language-dependent effects on readability. As shown in Figure 6, for the English task, readability was evaluated using the FKGL, where lower scores indicate easier readability. Across 25 paired responses, the mean FKGL changed from 10.20 (SD 1.58) for the original model to 10.35 (SD 0.81) for prompt + model, corresponding to a mean paired difference of 0.14 points. This difference was not statistically significant on paired Wilcoxon testing (V=189.5; *P*=.48), and the result was consistent in the paired *t* test (*t*_24_=0.58; *P*=.56). At the question level, 12 responses showed lower FKGL values after prompt optimization, whereas 13 showed higher values, indicating no consistent English readability gain. In the Chinese task, readability was evaluated using lexical richness, where lower values indicate easier readability. Across 25 paired responses, the mean lexical richness decreased from 5.25 (SD 0.14) for the original model to 5.03 (SD 0.08) for prompt + model, corresponding to a mean paired difference of –0.23 points. This improvement was statistically significant on paired Wilcoxon testing (V=0.0; *P*<.001) and remained significant in the paired *t* test (t_24_=–10.81; *P*<.001). All 25 Chinese responses showed lower lexical richness values after prompt optimization, indicating uniform improvement across the full Chinese question set. Taken together, these findings suggest that prompt optimization did not improve English readability but improved Chinese readability.

Prompt optimization significantly improved understandability in English but not in Traditional Chinese (Figure 7). In English, the mean PEMAT understandability score increased from 86.8 (SD 5.2) to 92.1 (SD 0.30), yielding a mean paired difference of 5.3 points (paired Wilcoxon signed rank test: V=150.0; *P*<.001). Understandability improved in 15 of 25 responses, worsened in 2, and was unchanged in 8. In Traditional Chinese, the mean score increased from 89.8 (SD 3.7) to 92.0 (SD 0.3), with a nonsignificant mean paired difference of 2.2 points (V=92.0; *P*=.07). Understandability improved in 8 responses, worsened in 7, and was unchanged in 10 responses. Notably, postoptimization PEMAT scores converged to approximately 92 with minimal variance (SD 0.30) in both languages; because rigid CRAFT format constraints generated structurally near-identical outputs, PEMAT demonstrated limited discriminatory capacity postoptimization, and the resulting near-zero-variance paired tests must be interpreted within this context.

**Figure 5:**
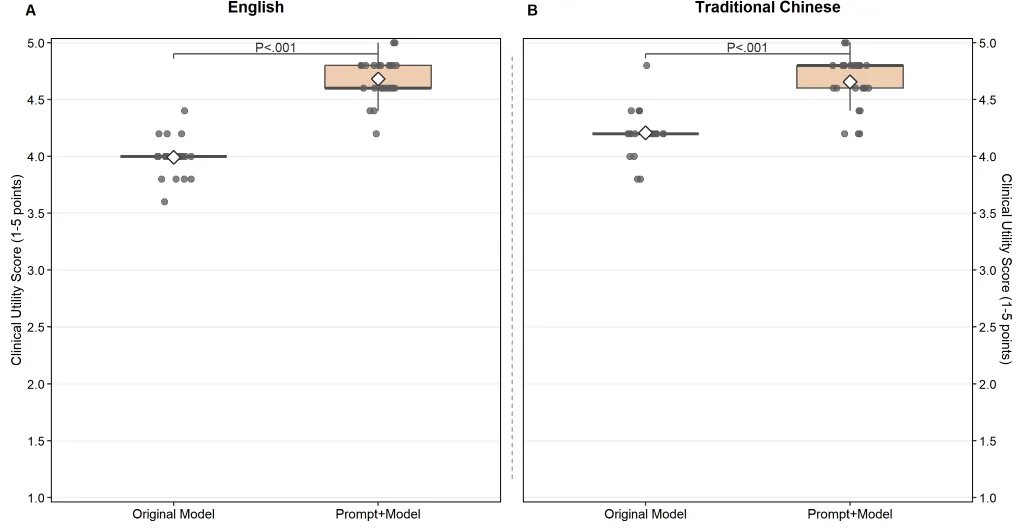
Comparison of response clinical utility scores for Gemini 3.1 Pro before and after prompt optimization. (A) English; (B) Traditional Chinese.

**Figure 6:**
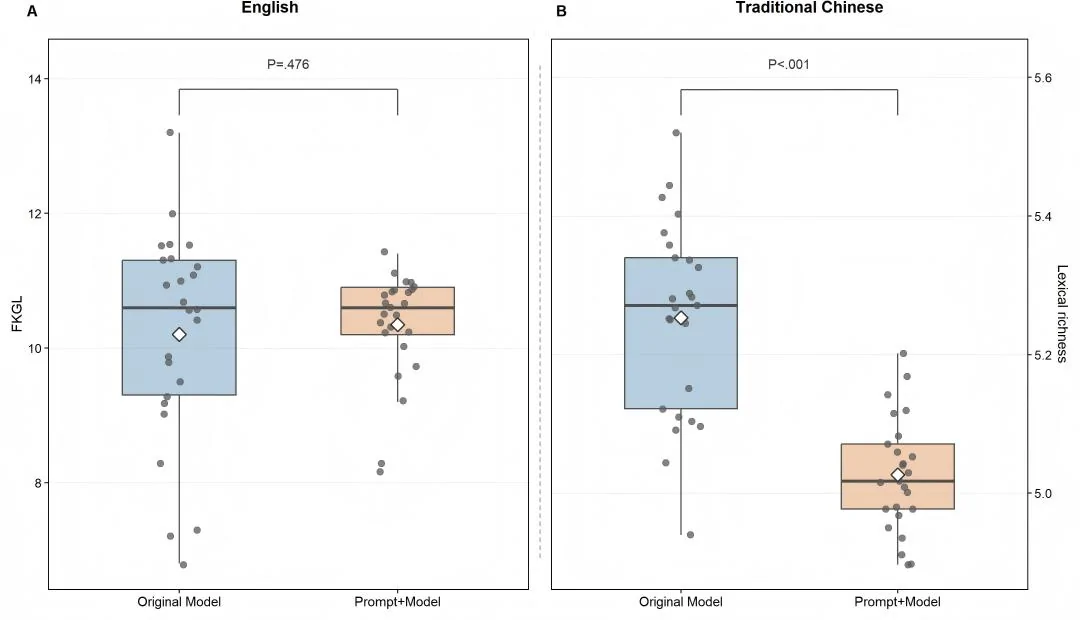
Readability before and after prompt optimization in English and Traditional Chinese: (A) English readability measured by Flesch-Kincaid Grade Level (FKGL) and (B) Traditional Chinese readability measured by lexical richness. Lower values indicate better readability in both panels.

**Figure 7:**
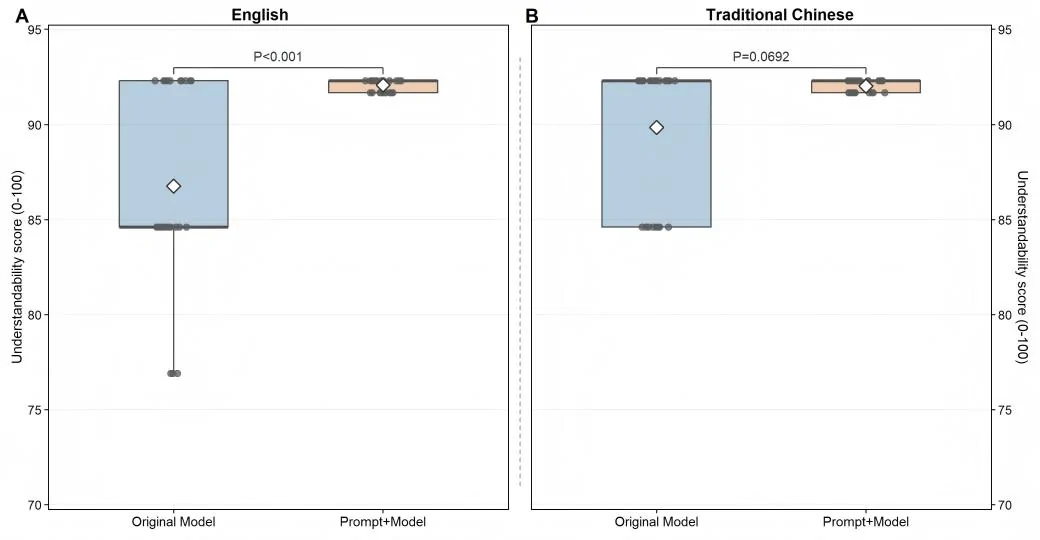
Question-level Patient Education Materials Assessment Tool (PEMAT) understandability scores before and after prompt optimization in (A) English responses and (B) Traditional Chinese responses. Each point represents 1 response. *P* values were calculated using paired Wilcoxon signed rank tests. Higher scores indicate better understandability.

### Reliability of the Evaluation Framework

Reliability of the evaluation framework was mixed. Agreement between the 5 expert raters on clinical utility was low (ICC=0.27, 95% CI 0.02-0.47), indicating substantial disagreement between clinicians applying a composite, partly normative construct; the linear mixed-effects models used for the domain-level analyses carry rater as a random intercept and therefore accommodate this variation, but it limits how far individual model rankings should be pressed. The independent checks were stronger. The blinded independent assessor’s safety classifications agreed with the panel’s consensus decisions on 248 responses (Cohen κ=0.94). The independent PEMAT rescoring of the random 20% subset agreed closely with the primary rater (ICC=0.90, 95% CI 0.88-0.92).

## Discussion

### Principal Findings

This 2-phase study evaluated 4 LLMs in Hong Kong’s bilingual cancer survivorship context. Gemini 3.1 Pro achieved the highest baseline clinical utility in both English (3.99/5) and Traditional Chinese (4.21/5). Despite higher overall clinical utility in Traditional Chinese, qualitative analysis revealed divergent safety failure typologies. Unsafe English responses (peaking at 24% in HK Chat-0.6.2) primarily involved contextual service mismatches and definitive symptom interpretations. Unsafe Traditional Chinese responses (reaching 20% in DeepSeek-V3.2) featured prescriptive chronic disease management and unverified adjunctive therapy recommendations. The most readable models were not the most clinically useful ones: DeepSeek-V3.2 and Kimi-K2.5 produced the most accessible texts in English and Traditional Chinese, respectively, while neither ranked highest for clinical utility in its language. Applying the CRAFT prompt to Gemini 3.1 Pro significantly improved clinical utility (English: 4.68; Traditional Chinese: 4.66) and resolved the 2 unsafe outputs observed in the English baseline without reducing understandability.

### Comparison With Prior Work

Recent evaluations across medical specialties confirm that while LLMs can synthesize comprehensive clinical information, their unconstrained outputs frequently harbor significant inaccuracies and safety risks [24,25]. Because most prior assessments focus strictly on monolingual English contexts [10], our bilingual evaluation identifies language-dependent differences in clinical safety. The divergence between English (contextual service mismatches) and Traditional Chinese (prescriptive recommendations for unverified adjunctive therapies) demonstrates that safety profiles fluctuate across a model’s linguistic settings. Monolingual validation remains clinically inadequate for patient-facing deployments in multilingual health care systems. Baseline comparisons also suggested a relationship between clinical utility and readability, although this was not formally tested. Within each language, the models producing the most accessible text were not those producing the most clinically useful text. This pattern is consistent with reports that guideline-adherent AI outputs tend to require higher reading grade levels because of the medical terminology involved, which may increase patient cognitive burden [26,27]. It should therefore be read as an observation in this purposive sample rather than as an inherent trade-off.

Our baseline clinical utility findings sit within a now-substantial body of oncology chatbot evaluations. A scoping review of 60 studies assessing the medical accuracy of LLMs in oncology found that question-answering as a health information resource was the single most common application and reported the same paired pattern we observed: usable accuracy alongside poor reliability, hallucination, and a continuing requirement for clinician oversight. That review’s principal recommendation was validation on external hold-out data [28]. A mean clinical utility score near 4 on a 5-point scale, as seen here at baseline, should therefore not be read as evidence of readiness for unsupervised patient-facing use.

The closest survivorship-specific comparator evaluated chatbot responses to the top unmet needs of adolescents and young adults with melanoma. It reported moderate information quality, stronger cognitive than emotional empathy, and a mean FKGL of 11.8, well above the level recommended for patient-facing material [29]. Our English model-level means spanned 8.4 to 15.8, a similar range, which suggests that the readability problem is a property of general-purpose chatbots rather than of any cancer population or question set. That study, like most in this literature, was conducted in English only. The present study adds a paired Traditional Chinese arm and treats safety as a separate outcome rather than inferring it from overall quality or fluency.

Our unsafe-response rates are broadly consistent with the wider safety literature. A physician-led red-teaming study of 4 publicly available chatbots found problematic responses in 21.6% to 43.2% of 888 responses to 222 patient-posed primary care questions, with frankly unsafe responses in 5% to 13% [30]. Our pooled unsafe rate of 10% (20/200) falls within that band despite a different clinical domain, a different rubric, and a bilingual design. What our data add is that a pooled rate conceals clinically important structure: the same model could be the safest in one language and the least safe in the other. DeepSeek-V3.2 produced no unsafe English responses but the highest unsafe rate in Traditional Chinese (5/25, 20%), whereas HK Chat-0.6.2 showed the opposite pattern (6/25, 24% in English; 1/25, 4% in Traditional Chinese). We identified no prior study that evaluated a single question set across both working languages of the same health system, and a single-language benchmark would have missed this asymmetry entirely.

The phase 2 result should be positioned cautiously against the prompt-engineering literature. The oncology scoping review documented a rapid increase in prompt-engineering and fine-tuning studies between 2022 and 2024 while emphasizing that external validation remains the outstanding requirement [28]. Because our CRAFT prompt was derived from errors observed in the same 25 questions on which it was subsequently tested, and was scored by the same panel, our finding is an exploratory within-sample effect that does not speak to generalizability. Reporting for the present study follows the CHART, developed specifically for chatbot health advice studies, which requires explicit reporting of model identifiers, model details, prompt engineering, and query strategy [16]. A direct numerical comparison with any of these studies remains limited because the comparator studies differ in model, prompt, rating instrument, language, and date of access.

The rubric we used was deliberately composite, and this shapes how the phase 2 result should be read. A maximum score of 5 required 3 things at once: factual correctness; an explicit, actionable safety warning where one was indicated; and guidance aligned with the Hong Kong health system, without requiring the reader to infer missing steps. A response about Chinese medicine for postchemotherapy fatigue might, for example, summarize the pharmacology correctly and still fail to warn about hepatotoxicity or to direct the reader to a registered Chinese medicine practitioner; under our rubric, that response is not clinically useful, whatever its factual content. Because the CRAFT prompt was written to enforce precisely the safety warnings and local specificity that the top of the rubric rewards, the improvement it produced should not be read as the model having acquired better medical knowledge. It is evidence that structured prompting can make an unchanged model conform to a clinical standard it does not meet by default.

Unconstrained commercial models carry clinical risks that necessitate structural limits. Expert-derived prompting (CRAFT) effectively restricted the model to a health information provider role, preventing overreaching medical management and hallucinated service data. Consistent with recent findings that structured instructions and retrieval-augmented architectures improve clinical adherence and reduce harm [26,31,32], the CRAFT framework demonstrates that contextually anchored, role-restrictive prompts are essential for safe, patient-facing oncology deployments.

### Limitations

Although this study provides valuable insights into the application of LLMs in cancer survivorship care, several limitations and corresponding future directions warrant consideration.

First, both the evaluation question list and the expert panel were relatively restricted in size. While the questions reflect authentic patient inquiries and the panel size aligns with published guidance [33], they may not capture the full complexity of real-world oncology consultations. Future research should use expanded question cohorts and larger expert panels, supplemented by standardized scoring calibration, to enhance representativeness and interrater reliability.

Second, temporal and practical constraints limited the assessment to models accessible in March 2026. Future longitudinal evaluations are essential to monitor the capabilities of newer iterations (eg, DeepSeek-V4 and GPT-5.5) and assess the long-term consistency and scalability of LLM-generated outputs.

Third, methodological constraints introduced potential biases. A language-of-origin effect may exist, as the original questions were sourced in Traditional Chinese. Consequently, the higher clinical utility scores for Chinese responses might partially reflect preserved cultural nuances rather than a fundamental superiority in Chinese-language processing. Furthermore, using consumer web interfaces precluded the disabling of automated internet searches. While this design ensures ecological validity by mirroring actual patient usage, it introduces retrieval-augmented generation as a confounding variable, confounding the models’ intrinsic parametric knowledge with real-time web-retrieval proficiency.

Fourth, phase 2 was exploratory rather than confirmatory. The CRAFT prompt was developed by the same expert panel, from errors identified in the same 25 questions, and was then evaluated on those same questions by the same raters. Phase 2 is therefore a within-sample comparison and not an independent validation, and it is open to overfitting and expectancy bias. Only the best-performing baseline model was tested with the prompt, so the result does not establish that the prompt would transfer to other models. A confirmatory study should lock the prompt on a development set and evaluate it on an independent hold-out set containing new, adversarial, urgent-symptom, and locally specific questions, across several models and interface configurations, with repeated independent runs, independent blinded raters, and prespecified paired analyses reporting effect estimates with uncertainty intervals.

Fifth, the measurement properties of our outcomes are limited. The 5-point clinical utility rubric was expert-derived and composite: its higher categories reward the presence of safety warnings and local actionability as well as factual correctness, so it overlaps conceptually with the separate safety outcome and with the very features the CRAFT prompt was designed to add. This overlap may inflate the observed phase 2 difference, and the score should be read as composite clinical usefulness rather than as factual accuracy alone. Agreement between the 5 expert raters was low (ICC=0.27), which limits how far individual model rankings should be pressed, even though the mixed-effects models used for the primary analyses carry rater as a random effect. Safety was a single panel consensus judgment rather than independent duplicate coding, and PEMAT was scored by a single rater; both were checked against an independent blinded assessor during revision, but a check made after the fact is weaker than duplicate coding built into the original design. Prespecifying and separately validating factual accuracy, safety, local actionability, and communication as distinct constructs is a requirement for the confirmatory study described above.

Sixth, only 1 initial response was recorded for each model-question-language condition. Commercial chatbots are stochastic and are updated continuously, and we neither regenerated responses nor repeated queries on separate occasions, so within-condition variability cannot be characterized. Our findings describe the outputs sampled during the study window and should not be treated as stable estimates of model behavior.

Seventh, our readability measures are indirect and not comparable across languages. FKGL estimates an English school grade from sentence and word length, whereas the AlphaReadabilityChinese 1.0 index is a language-specific lexical statistic. The two are constructed differently, sit on different scales, and neither measures comprehension by survivors of cancer in Hong Kong. We therefore restricted readability comparisons to models within the same language and did not test the association between clinical utility and readability.

Future work should prioritize multiturn and adversarial testing, larger and more diverse question sets, and newer model releases, and should use API-driven ablation designs to separate the contribution of live web retrieval from parametric knowledge. Two further studies follow directly from the limitations above. The first would establish the construct validity of the clinical utility rubric, using exploratory and confirmatory factor analysis to test whether factual correctness, safety, local actionability, and communication behave as separable dimensions. The second would recruit a local cohort of survivors of cancer to assess criterion validity, testing model outputs against validated health literacy instruments and against behavioral end points rather than against expert judgment alone. Advanced optimization strategies such as fine-tuning and robust regulatory oversight of AI-generated health information remain parallel requirements for responsible implementation.

### Conclusions

Unconstrained commercial LLMs exhibit variable clinical utility and substantial clinical safety risks when addressing bilingual cancer survivorship inquiries in Hong Kong. While Google Gemini 3.1 Pro demonstrated the highest baseline clinical utility, safety vulnerabilities remained highly language dependent. English outputs were predominantly compromised by contextual service mismatches, whereas Traditional Chinese outputs frequently generated prescriptive medical management and endorsed unverified adjunctive therapies. Furthermore, within each language, the models generating the most readable texts were not those achieving the highest clinical utility, although this relationship was not formally tested. In an exploratory within-sample comparison, an expert-designed CRAFT prompting strategy improved clinical utility and resolved the unsafe recommendations observed at baseline without reducing understandability; this result requires confirmation on an independent question set. Deploying LLMs for oncological support in multilingual systems therefore mandates rigorous, role-restrictive prompting frameworks to ensure clinical safety.

## Appendix

Multimedia Appendix 1 Question list.

Multimedia Appendix 2 CHART (Chatbot Assessment Reporting Tool) checklist.

Multimedia Appendix 3 The structured context, role, audience, format, task, and tone (CRAFT) prompts.

Multimedia Appendix 4 The scoring scale of response clinical utility.

Multimedia Appendix 5 Phase 1 results.

Multimedia Appendix 6 Phase 2 results.

## Abbreviations

AHRQ: Agency for Healthcare Research and Quality
CHART: Chatbot Assessment Reporting Tool
CRAFT: context, role, audience, format, task, and tone
FKGL: Flesch-Kincaid Grade Level
ICC: intraclass correlation coefficient
LLM: large language model
PEMAT: Patient Education Materials Assessment Tool

## Data Availability

The study was not prospectively registered, and no protocol was published in advance. All data generated or analyzed during this study are included in this published article and its supplementary information files. The question set, the CRAFT prompts, the clinical utility scale, and the phase 1 and phase 2 result tables are provided as Multimedia Appendices 1, 3-6. The complete records of model responses are available from the corresponding author on reasonable request.
